# Heme oxygenase-1 prevents murine intestinal inflammation

**DOI:** 10.3164/jcbn.17-133

**Published:** 2018-07-12

**Authors:** Tomohisa Takagi, Yuji Naito, Katsura Mizushima, Yasuko Hirai, Akihito Harusato, Tetsuya Okayama, Kazhuhiro Katada, Kazuhiro Kamada, Kazuhiko Uchiyama, Osamu Handa, Takeshi Ishikawa, Yoshito Itoh

**Affiliations:** 1 Molecular Gastroenterology and Hepatology, Graduate School of Medical Science, Kyoto Prefectural University of Medicine, 465 Kajii-cho, Kawaramachi-Hirokoji, Kamigyo-ku, Kyoto 602-8566, Japan; 2 Department for Medical Innovation and Translational Medical Science, Graduate School of Medical Science, Kyoto Prefectural University of Medicine, 465 Kajii-cho, Kawaramachi-Hirokoji, Kamigyo-ku, Kyoto 602-8566, Japan

**Keywords:** heme oxygenase-1 (HO-1), BTB and CNC homolog 1 (Bach1), dextran sodium sulfate (DSS)-induced colitis

## Abstract

Heme oxygenases (HOs) are rate-limiting enzymes catabolizing heme to biliverdin, ferrous iron, and carbon monoxide, and of the three HO isoforms identified, HO-1 plays a protective role against inflammatory processes. In this study, we investigated the possible role of HO-1 in intestinal inflammation. Acute colitis was induced in male C57BL/6 (wild-type) and homozygous BTB and CNC homolog 1 (Bach1)-deficient mice, which show high HO-1 expression in the colonic mucosa, using dextran sodium sulfate. The disease activity index, myeloperoxidase activity, and inflammatory cytokines in the colonic mucosa were evaluated 7 days after dextran sodium sulfate-dependent colitis induction. We also evaluated the impact of HO-1 inhibition using zinc protoporphyrin IX (25 mg/kg i.p., daily). After dextran sodium sulfate administration, HO-1 mRNA and protein expression increased in a time-dependent manner. Disease activity index score, myeloperoxidase activity, and colonic production of TNF-α and IFN-γ were increased after dextran sodium sulfate administration, and co-administration of zinc protoporphyrin IX enhanced their increase. In addition, disease activity index in Bach1-deficient was significantly lower after dextran sodium sulfate administration than that in wild type mice. These results indicate that HO-1 plays a protective role against dextran sodium sulfate-induced intestinal inflammation, possibly by regulating pro-inflammatory cytokines in intestinal tissues.

## Introduction

Inflammatory bowel disease (IBD), including ulcerative colitis (UC) and Crohn’s disease (CD), is a chronic and relapsing intestinal inflammatory disorder. Although the precise pathogenesis of IBD remains unknown, intestinal mucosal immunity, genetics, microbiome, and environment play an important role in disturbing intestinal homeostasis, leading to the development and perpetuation of intestinal inflammation.^(1,2)^ In addition, recent evidences from clinical and basic science studies reveal that intestinal inflammation is associated with an imbalance between increased oxidative stress and decreased antioxidant activity, which may explain at least in part, several aspects of the clinical and pathophysiological features of IBD patients.^(3–5)^

One of the antioxidant enzymes, heme oxygenase (HO), is a rate-limiting enzyme catalyzing the degradation of heme to biliverdin, free iron, and carbon monoxide (CO).^(6–8)^ In particular, HO-1 is one of three mammalian HO isozymes and is a stress-responsive protein induced by various stimuli including oxidative stress, ischemia-reperfusion (I/R), heavy metals, cytokines, and heme (its substrate). Several transcriptional regulatory proteins are involved in the regulation of HO-1 expression including nuclear factor erythroid 2-related factor-2 (Nrf2) and the BTB and CNC homolog 1 (Bach1). Nrf2 positively regulates HO-1 transcription, whereas Bach1 competes with Nrf2 and represses transcription.^(6,9,10)^

Current research efforts are focused on understanding the role of HO-1 in intestinal inflammation. HO-1 is expressed at very low levels in the gastrointestinal tract under normal conditions, but its expression is induced upon gastrointestinal mucosal injury and/or inflammation. We have previously revealed that HO-1 expression in the inflamed colonic mucosa of patients with active UC is strongly increased compared to the normal colonic mucosa.^(11,12)^ In addition, in studies using murine experimental colitis models, HO activity and HO-1 expression are markedly increased with the development of colitis, and inhibition of HO activity potentiates colonic damage and inflammation.^(13,14)^ Thus, induction of HO-1 expression in the intestinal mucosa might inhibit the development of intestinal inflammation; however, further evidence is needed to support this advantageous effect of HO-1.

The present study aimed to investigate the protective role of HO-1 in a murine dextran sodium sulfate (DSS)-induced colitis model, which shares both immunological and pathological features with human IBD. In addition, we investigated the role of HO-1 in intestinal inflammation using Bach1-deficient mice, which show high HO-1 expression in the colonic mucosa.

## Materials and Methods

### Animals

Seven-week old male C57BL/6 mice were purchased from SHIMIZU Laboratory Supplies Co., Ltd. (Kyoto, Japan). Bach1-deficient mice were a gift from Prof. Kazuhiko Igarashi (Tohoku University, Sendai, Japan). Animal maintenance and experimental procedures were performed in accordance with the National Institutes of Health (NIH) guidelines for the use of experimental animals. All procedures were approved by the Animal Care Committee of Kyoto Prefectural University of Medicine (Kyoto, Japan).

### Induction of colitis

Acute colitis was induced by administering 2.0% (w/v) DSS (Wako Pure Chemical Industries, Ltd., Osaka, Japan) orally in drinking water *ad libitum* for 7 days as described in previous studies.^(15–17)^ The HO inhibitor, zinc protoporphyrin (ZnPP; Frontier Scientific, Logan, UT) at 25 mg/kg, was injected intraperitoneally daily during DSS administration. The mice were sacrificed on day 7 and their colons were removed for macroscopic, histological, and biochemical examination.

### Evaluation of colitis severity

We evaluated the disease activity index (DAI), colon length, and histology. The DAI was determined by scoring the changes in animal weight, occult blood positivity, gross bleeding, and stool consistency, as described previously.^(15–17)^ We used five grades of weight loss (0, no loss or weight gain; 1, 1–5% loss; 2, 5–10% loss; 3, 10–20% loss; 4, >20% loss), three grades of stool consistency (0, normal; 2, loose; and 4, diarrhea), and three grades of occult blood (0, negative; 2, occult blood-positive; and 4, gross bleeding).

After determining the DAI for 7 days, mice were sacrificed, the entire colon between the cecum and the anus was removed, and colon length was measured as an indirect marker of inflammation. The distal colon was fixed in 10% buffered formalin for histological analysis. Sections of 5-µm thickness were prepared and stained with hematoxylin and eosin (H&E). Slides were then examined and scored in a blinded fashion using a previously published grading system.^(16,18,19)^ Briefly, histology was scored as follows: Epithelium (E) 0: normal morphology, 1: loss of goblet cells, 2: loss of goblet cells in large areas, 3: loss of crypts, and 4: loss of crypts in large areas; Infiltration (I) 0: no infiltration, 1: infiltration around crypt bases, 2: infiltration reaching the muscularis mucosa, 3: extensive infiltration reaching the muscularis mucosa and thickening of the mucosa with abundant edema, and 4: infiltration of the submucosa. The total histological score represents the sum of the epithelium and infiltration scores, and thus ranges from 0 to 8 (total score = E + I).

### Assessment of HO-1 expression and localization in inflamed colonic mucosa

For quantification of *ho-1* and *β-actin* mRNA as an internal control, total RNA was isolated from intestinal mucosal tissue using the acid guanidinium phenol chloroform method with an Isogen kit (Nippon Gene Co., Ltd., Toyama, Japan), and RNA concentration was determined by absorbance at 260 nm in relation to that at 280 nm. The RNA was used for amplification by reverse transcription polymerase chain reaction in a 10 µl mixture containing 2 µl of the reverse transcriptase product, 0.1 µM of both the sense and anti-sense primers, 0.2 mM dNTP mix, and 0.5 U Taq DNA polymerase (TaKaRa Bio, Inc., Otsu, Shiga, Japan). The reaction was performed as follows: 30 cycles of amplification (denaturation at 94°C for 30 s, annealing at 56°C for 30 s, and extension at 72°C for 45 s), followed by a final extension step of 7 min at 72°C.

The following primer sequences were used: for *ho-1*, sense 5'-TGGGTCCTCACTCTCAGCTT-3', and antisense 5'-GTCGTG GTCAGTCAACATGG-3'; and for *β-actin*, sense 5'-TGGAAT CCTGTGGCATCCA-3', and antisense 5'-TAACAGTCCGCC TAGAAGCA-3'.

The polymerase chain reaction products were separated electrophoretically on a 2.5% agarose gel and stained with ethidium bromide.

The total protein from colonic mucosa was mixed with SDS sample buffer. The samples were subjected to 12% SDS-PAGE and blotted onto a nitrocellulose membrane. The membrane was probed using rabbit polyclonal anti-HO-1 antibody (1:1,000 dilution in TBS-T) and goat polyclonal anti-actin (1:1,000) at room temperature for 1 h. After three washes with TBS-T, the membrane was incubated with anti-rabbit IgG-HRP (1:3,000; GE Healthcare, Piscataway, NJ) at room temperature for 1 h. The immunoreactive proteins were visualized using the ECL Plus Western Blotting Detection System (GE Healthcare).

Double labeling with immunofluorescence staining was performed and visualized using a laser scanning confocal microscope (FV10i; Olympus, Tokyo, Japan) to examine the co-localization of HO-1 and the macrophage marker, F4/80 in the inflamed mucosa. Serial 4 µm-thick cryostat sections were mounted on silanized slides and incubated overnight at 4°C with a rabbit monoclonal antibody specific to HO-1 (Stressgen, Brussels, Belgium) and a rat monoclonal antibody specific to F4/80 (Abcam, Cambridge, MA). The secondary antibodies, chicken anti-rabbit immunoglobulin G (IgG) labeled with Alexa Fluor 594 (Invitrogen, Carlsbad, CA) or donkey anti-rat IgG labeled with Alexa Fluor 488 (Invitrogen) were then added to bind to the primary antibodies.

### Measurement of MPO activity

Tissue-associated myeloperoxidase (MPO) activity was determined by a modification of the method by Grisham *et al.*^(20)^ as an index of neutrophil accumulation. Mucosal homogenates (2 ml) were centrifuged at 20,000 × *g* for 15 min at 4°C to pellet the insoluble cellular debris. The pellet was then rehomogenized in an equivalent volume of 0.05 mol potassium phosphate buffer (pH 5.4) containing 0.5% hexadecyltrimethylammonium bromide. The samples were centrifuged at 20,000 × *g* for 15 min at 4°C, and the supernatants were collected. MPO activity was assessed by measuring the H_2_O_2_-dependent oxidation of 3,3',5,5'-tetramethylbenzidine. One unit of enzyme activity was defined as the amount of MPO that caused a change in absorbance of 1.0/min at 460 nm and 37°C. The total protein content in the tissue homogenates was measured using a Bio-Rad Protein Assay kit (Bio-Rad Laboratories, K.K., Tokyo, Japan) according to the manufacturer’s protocol.

### Determination of intestinal concentration of tumor necrosis factor (TNF)-α and interferon (IFN)-γ

We determined the concentration of TNF-α and IFN-γ in the supernatant of mucosal homogenates using an enzyme-linked immunosorbent assay (ELISA) kit (eBioscience Inc., CA). The assay was performed according to the respective manufacturer’s instructions. After color development, optical densities were measured at 450 nm using a plate reader (Spectramax M2; Molecular Devices Corp. Sunnyvale, CA).

### Statistical analysis

The results are presented as the mean ± SEM. Overall differences between the groups were determined by one-way analysis of variance. When the one-way analysis of variance was significant, differences between individual groups were analyzed using the Bonferroni’s multiple comparisons test. Differences with *p*<0.05 were considered significant. All analyses were performed using the GraphPad Prism 6 program (GraphPad Software Inc., San Diego, CA) for Macintosh.

## Results

### HO-1 expression in colonic mucosa

**Fig. 1:**
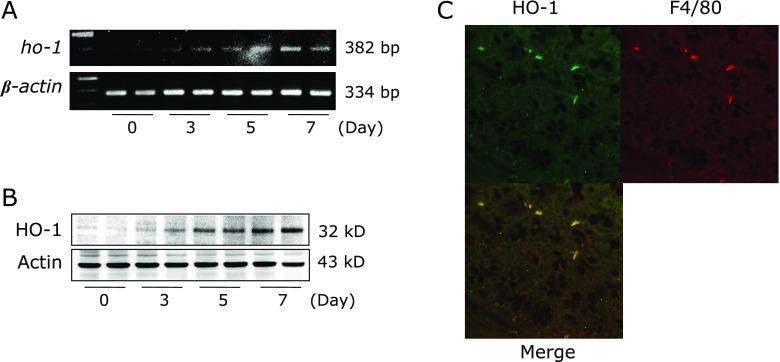
Effect of zinc protoporphyrin (ZnPP) on DSS-induced colitis. To analyze HO-1 expression in colonic mucosa, time-dependent expression of *ho-1* mRNA in colonic mucosal tissue after DSS administration was determined using RT-PCR (A). Time-dependent expression of HO-1 protein in colonic mucosa after DSS administration was determined by western blotting (B). The results representative of 3 separate experiments. Each treatment condition after DSS treatment is represented by 2 lanes. (C) Colonic Localization of HO-1 expression was analyzed by immunofluorescence staining and visualized under a laser scanning confocal microscope. Upper left: HO-1 stained with anti-rabbit Alexa 594 secondary antibody; Upper right: F4/80 stained with anti-rat Alexa 488 secondary antibody; Below: merged image of upper images. A representative image from three separate experiments is shown.

The expression of *ho-1* mRNA in murine colonic mucosa was increased in a time-dependent manner after DSS administration (Fig. 1A). The results of western blot analysis showed that HO-1 protein expression in the colonic mucosa was markedly increased after DSS administration, in accord with the result of *ho-1* mRNA expression (Fig. 1B). Thus, HO-1 expression was increased in association with the development of intestinal inflammation.

Furthermore, to investigate the localization of HO-1 expression in the inflamed colonic mucosa, we performed immunofluorescence staining. As depicted in Fig. 1C, HO-1-positive cells were observed in the inflamed colonic mucosal tissue. Merged images showed that the observed HO-1-positive cells were mostly F4/80-immunopositive macrophages.

### Effects of HO-1 inhibition on intestinal inflammation

**Fig. 2:**
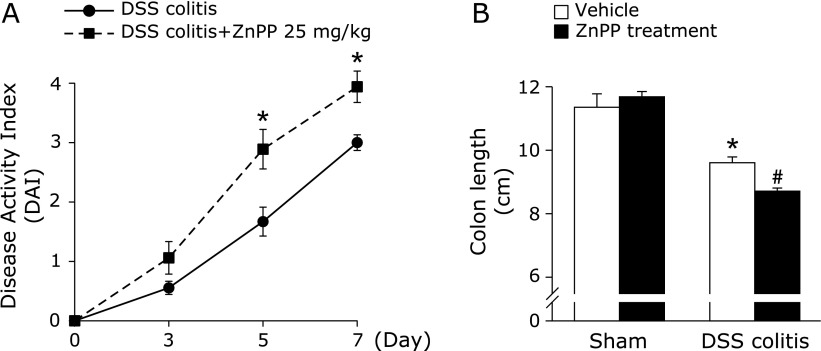
Effects of HO-1 inhibition on dextran sulfate sodium (DSS)-induced colitis. (A) The effect of ZnPP on the disease activity index during the development of dextran sulfate sodium (DSS)-induced colitis in mice. Each value indicates the mean ± SE for 8 mice. *p<0.05 compared with control mice receiving 2% DSS solution alone. (B) The effect of ZnPP on the total length of the colon after DSS administration. Each value indicates the mean ± SE for 8 mice. *p<0.05 compared with sham mice receiving vehicle, ^#^*p*<0.05 compared with control mice receiving 2% DSS solution alone.

Mice exposed to 2% DSS developed symptoms of acute colitis with diarrhea followed by rectal bleeding and severe weight loss. The DAI scores in DSS-treated mice determined by weight loss, stool consistency, and blood in stool, were significantly increased in ZnPP-treated mice than in the controls (significant from day 5; Fig. 2A). As shown in Fig. 2B, the colon length was significantly decreased at 7 days after DSS administration. This reduction in colon length was significantly enhanced by ZnPP treatment.

**Fig. 3:**
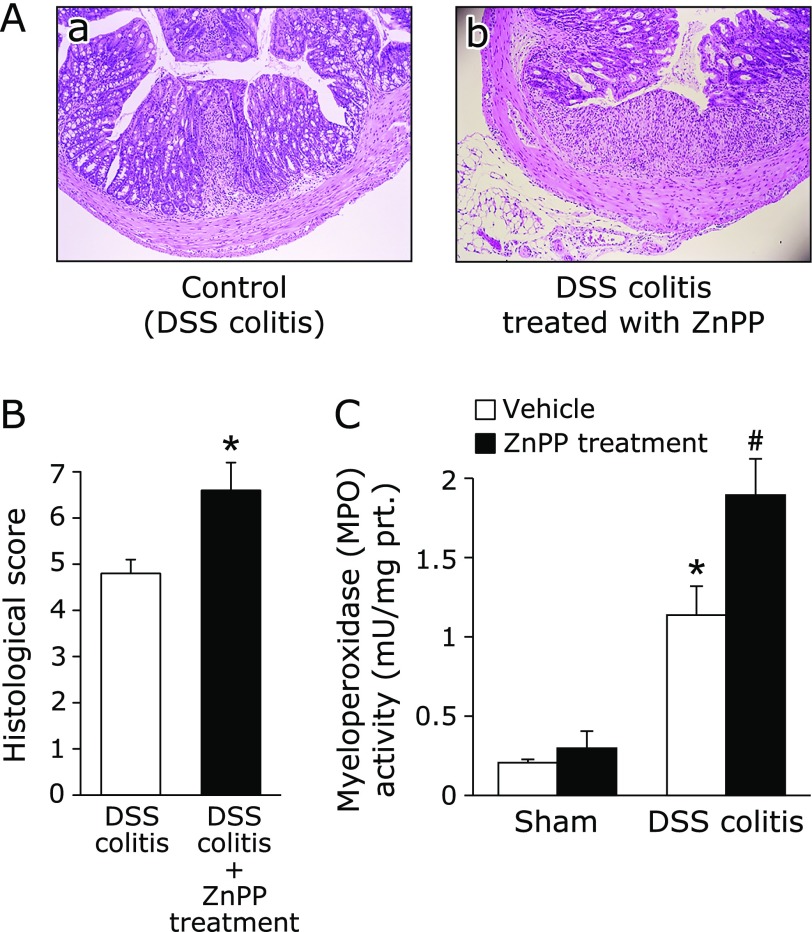
Deterioration of colitis by HO-1 inhibition. (A) The histological appearance of colonic tissue in mice with DSS induced-colitis receiving vehicle (a) and those treated with ZnPP (b). Loss and shortening of crypts, mucosal erosions, inflammatory cell infiltration, and goblet cell depletion are seen in (a). In (b), larger erosions are associated with much inflammatory cell infiltration. Hematoxylin and eosin staining. Magnification, ×40. (B) Histological score was evaluated as described in Materials and Methods. The data represent the mean ± SE of 5 mice. *p<0.05 compared with mice with DSS induced-colitis receiving vehicle. (C) Effect of ZnPP on neutrophil accumulation expressed as myeloperoxidase (MPO) activity. Each value indicates the mean ± SE for 7 mice. *p<0.05 compared with mice receiving vehicle, ^#^*p*<0.05 compared with control mice receiving 2% DSS solution alone.

The effect of HO-1 inhibition was also confirmed by a histological study. Fig. 3A shows the typical histological appearances in control mice (DSS colitis) and in the ZnPP-treated group. Administration of 2% DSS alone for 7 days resulted in large areas showing epithelial crypt loss, prominent neutrophil infiltration throughout the mucosa, ulceration, and mucosal bleeding. Treatment with ZnPP resulted in larger erosions with more neutrophils. The histological scores reflect these findings (Fig. 3B). Neutrophil accumulation was evaluated by measuring the tissue-associated MPO activity in colonic mucosa homogenates. The increase in colonic mucosa MPO activity after DSS administration was markedly increased by ZnPP treatment (Fig. 3C). Thus, inhibition of HO-1 by ZnPP, an HO-1 inhibitor, obviously aggravated intestinal inflammation.

### Effects of HO-1 inhibition on mucosal concentrations of TNF-α and IFN-γ

**Fig. 4:**
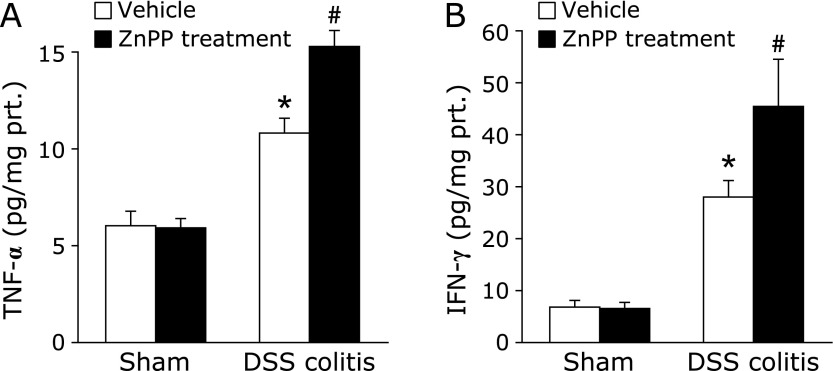
Effect of ZnPP on tumor necrosis factor (TNF)-α (A) and interferon (IFN)-γ (B) in the colonic mucosa of mice. Each value indicates the mean ± SE for 7 mice. *p<0.05 compared with mice receiving the vehicle, ^#^*p*<0.05 compared with control mice receiving 2% DSS solution alone.

To investigate whether ZnPP treatment aggravated the inflammatory response through cytokine regulation, we analyzed the intestinal levels of TNF-α and IFN-γ. A substantial increase in mucosal TNF-α and IFN-γ concentration was found in the control mice on day 7 of DSS administration (Fig. 4A and B). These increases were significantly enhanced by ZnPP treatment.

### Bach1 deficiency ameliorated DSS-induced colitis

**Fig. 5:**
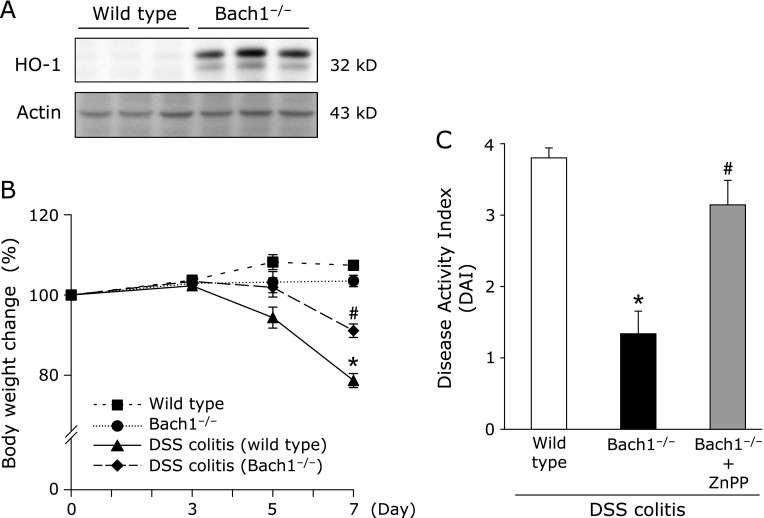
Bach1 deficiency ameliorated DSS-induced colitis. (A) HO-1 protein expression in the colonic mucosa of Bach1-deficient mice was determined by western blotting. Results are representative of 3 separate experiments. (B) The degree of body weight loss during DSS treatment. Each value indicates the mean ± SE for 6 mice. *p<0.05 compared with wild type mice receiving vehicle, ^#^*p*<0.05 compared with wild type mice receiving 2% DSS solution. (C) Disease activity index (DAI) on day 7 of DSS treatment. The effect of ZnPP on DAI during the development of DSS-induced colitis in Bach1-deficient mice was also evaluated. Each value indicates the mean ± SE for 5 mice. *p<0.05 compared with wild type mice receiving 2% DSS solution, ^#^*p*<0.05 compared with Bach1-deficient mice receiving 2% DSS solution.

In Bach1-deficient mice, HO-1 protein expression in the colonic mucosa was markedly increased compared with that in wild-type mice (Fig. 5A). The degree of body weight loss after DSS administration was also inhibited in Bach1-deficient mice compared with that in wild-type mice (Fig. 5B). The increased DAI score in wild-type mice 7 days after DSS administration was also significantly inhibited in Bach1-deficient mice. Moreover, this inhibitory effect on colitis in Bach1-deficient mice was cancelled by co-treatment with the HO-1 inhibitor, ZnPP (Fig. 5C).

**Fig. 6:**
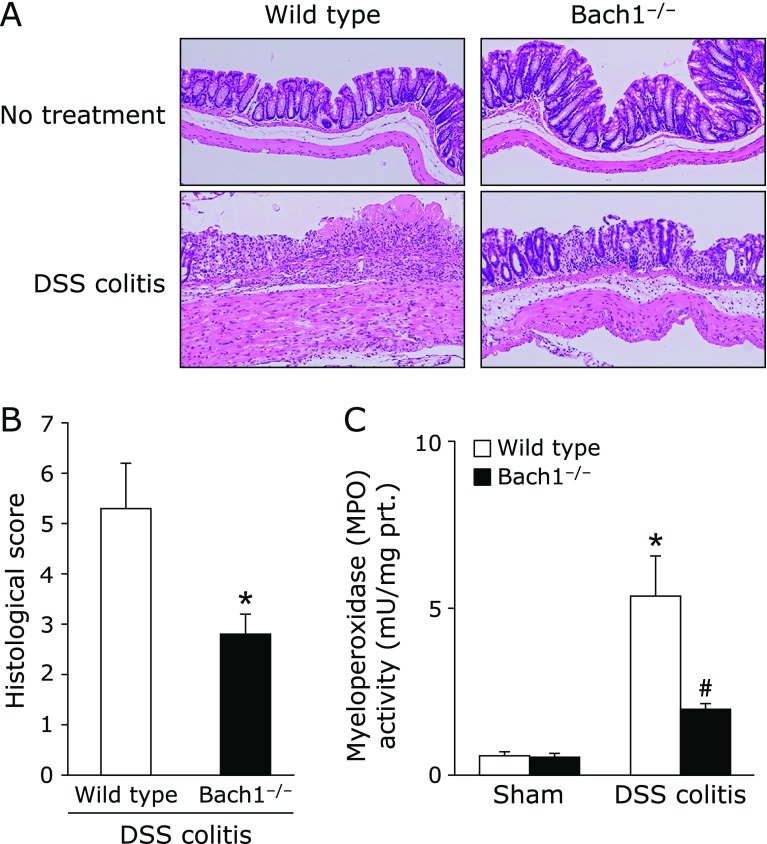
Bach1 deficiency ameliorated DSS-induced colitis. (A) The histological appearance of colonic tissue in wild type mice and Bach1-deficient mice without or with DSS induced-colitis. Hematoxylin and eosin staining. Magnification, ×40. (B) Histological score was evaluated as described in Materials and Methods. Data represent the mean ± SE of 5 mice. *p<0.05 compared to wild type mice with DSS induced-colitis. (C) Tissue-associated neutrophil accumulation in the colonic mucosa expressed as myeloperoxidase (MPO) activity. Each value indicates the mean ± SE for 7 mice. *p<0.05 compared with wild type mice without 2% DSS solution, ^#^*p*<0.05 compared with wild type mice receiving 2% DSS solution.

Histological examination revealed extensive ulceration with inflammatory cell infiltration after DSS administration. In contrast, the extent of mucosal damage and inflammatory cell infiltration was markedly inhibited in Bach1-deficient mice (Fig. 6A). The histologic scores of Bach1-deficient mice were also significantly decreased than those of wild-type mice (Fig. 6B). Neutrophil accumulation was evaluated by measuring tissue-associated MPO activity in colonic mucosal homogenates (Fig. 6C). MPO activity was significantly suppressed in Bach1-deficient mice compared with that in wild-type mice.

## Discussion

In the present study, we demonstrated that inhibition of HO-1 aggravated intestinal inflammation in a model of DSS-induced colitis. These results were in agreement with those of previous studies.^(14)^ In addition, Bach1-deficient mice, which show increased HO-1 expression in the colonic mucosa, showed inhibition of intestinal inflammation development. Importantly, these protective effects in Bach1-deficient mice were abolished by treatment with an HO-1 inhibitor (Fig. 5C). Thus, HO-1 conferred protection from the development of intestinal inflammation.

In intestinal inflammation such as in active UC, HO-1 expression is increased compared to that in the non-inflamed colonic mucosa.^(11,12)^ We confirmed that HO-1 is mainly localized in CD68-positive macrophages in the colonic submucosal layer, though several other investigations have reported that HO-1 is expressed in inflammatory cells and in epithelial cells.^(21,22)^ Harusato *et al.*^(23)^ demonstrated that HO-1 expression in Bach1-deficient mice was localized mainly in F4/80 and CD11b macrophages. Importantly, these high HO-1-expressing macrophages presented M2-type markers, indicating an anti-inflammatory function. Further experiments indicated that transfer of these macrophages into wild-type mice, inhibited TNBS-induced colitis. Thus, HO-1 expression in macrophages induces an anti-inflammatory action.

Considering the mechanism by which induction of endogenous HO-1 expression can confer anti-inflammatory effects in intestinal inflammation, the pharmacological application of HO-1 end-products, particularly CO, can mimic the HO-1-dependent anti-inflammatory action. In fact, exogenous CO suppresses TNBS-induced colitis through inhibition of TNF-α production.^(24)^ Similarly, the beneficial effects of CO and HO-1 induction were also demonstrated in the models of chronic Th1-mediated colitis in IL-10-deficient mice^(25)^ and Th2-mediated colitis in TCRα-deficient mice.^(26)^

In summary, the present study indicates that HO-1 plays a protective role against the intestinal inflammation. However, further studies are required to elucidate the detailed mechanisms involved in this protective effect on the inflamed mucosa through HO-1. HO-1 may have great potential as a new therapeutic target for treatment of patients with IBD.
